# A Shift in Perspective: A Role for the Type I Toxin TisB as Persistence-Stabilizing Factor

**DOI:** 10.3389/fmicb.2022.871699

**Published:** 2022-03-17

**Authors:** Daniel Edelmann, Bork A. Berghoff

**Affiliations:** Institute for Microbiology and Molecular Biology, Justus Liebig University Giessen, Giessen, Germany

**Keywords:** toxin-antitoxin systems, *tisB/istR-1*, type I toxins, persistence, awakening, fluoroquinolones, SOS response

## Abstract

Bacterial persistence is a phenomenon that is founded by the existence of a subpopulation of multidrug-tolerant cells. These so-called persister cells endure otherwise lethal stress situations and enable restoration of bacterial populations upon return to favorable conditions. Persisters are especially notorious for their ability to survive antibiotic treatments without conventional resistance genes and to cause infection relapse. The persister state is typically correlated with reduction or inhibition of cellular activity. Early on, chromosomal toxin-antitoxin (TA) systems were suspected to induce the persister state in response to environmental stress. However, this idea has been challenged during the last years. Especially the involvement of toxins from type II TA systems in persister formation is put into question. For toxins from type I TA systems the debate has just started. Here, we would like to summarize recent knowledge gained for the type I TA system *tisB/istR-1* from *Escherichia coli*. TisB is a small, membrane-targeting toxin, which disrupts the proton motive force (PMF), leading to membrane depolarization. Based on experimental data, we hypothesize that TisB primarily stabilizes the persister state through depolarization and further, secondary effects. We will present a simple model that will provide a framework for future directions.

## Introduction

Toxin-antitoxin (TA) systems are genetic modules that are frequently found on bacterial chromosomes and plasmids (Pandey and Gerdes, 2005; Leplae et al., 2011; Harms et al., 2018; Srivastava et al., 2021; Jurėnas et al., 2022). TA systems typically consist of two factors: a toxin protein that has an inhibitory or poisonous effect on the host organism and an antitoxin that either counteracts expression of the toxin or antagonizes toxin activity (Page and Peti, 2016; Harms et al., 2018). However, under particular stress conditions, the toxin may overcome the neutralizing effect of its cognate antitoxin, which eventually leads to a targeted inhibition or poisoning of an essential biosynthetic process. Various aspects of the bacterial lifestyle are associated with expression of toxins from TA systems including (i) genome stability, (ii) phage defense, (iii) stress adaptation, and (iv) persister formation (Wang and Wood, 2011; Page and Peti, 2016; Harms et al., 2018; Fraikin et al., 2020; Song and Wood, 2020; Jurėnas et al., 2022).

Different TA system types have been described over the years, and we would like to refer the reader to recent reviews on TA system biology (Srivastava et al., 2021; Jurėnas et al., 2022). For the scope of this *Perspective*, we would like to focus on type I TA systems. A hallmark of type I TA systems is the existence of an RNA antitoxin that confers translational inhibition on the toxin mRNA (Gerdes and Wagner, 2007; Brantl and Jahn, 2015; Berghoff and Wagner, 2017). Antitoxin and toxin transcripts are often *cis*-encoded by overlapping genes, while transcription depends on the activity of individual promoters (Fozo et al., 2008; Brantl and Jahn, 2015). Alternatively, some type I TA systems are arranged with non-overlapping genes, but the transcripts show sufficient complementarity for duplex formation when encoded in *trans*. *Via* duplex formation, antitoxin RNAs render the ribosome binding site (canonical or standby) of toxin mRNAs inaccessible and suppress translational (e.g., Thisted and Gerdes, 1992; Darfeuille et al., 2007; Jahn and Brantl, 2013; Wen et al., 2017). The duplex is typically cleaved and/or degraded by cellular RNases. In addition to control by RNA antitoxins, secondary structures in the 5′ untranslated region (UTR) render the toxin primary transcripts translationally inactive, resulting in transcription-translation uncoupling (Berghoff and Wagner, 2017; Masachis and Darfeuille, 2018). This sophisticated post-transcriptional regulation by both mRNA secondary structures and RNA antitoxins likely avoids toxin production during growth-promoting, non-stress conditions. However, elevated transcription of the toxin gene (e.g., upon stress), and subsequent processing of the primary transcript into a translationally active mRNA, might eventually shift the ratio between toxin mRNA and RNA antitoxin in favor of the toxin mRNA. Hypothetically, excess toxin mRNA only occurs in a fraction of cells, and only these cells will produce sufficient toxin amounts to be physiologically affected (Berghoff and Wagner, 2019). Therefore, the regulatory features of type I TA systems favor phenotypic heterogeneity of clonal populations (Berghoff et al., 2017a). Type I toxins are typically small (<50 amino acids), membrane-targeting proteins that are frequently associated with disruption of the proton motive force (PMF) and/or the cytoplasmic membrane (Brielle et al., 2016). The concomitant growth inhibition in a fraction of cells is clearly consistent with the phenomenon of bacterial persistence. Persistence is caused by a subpopulation of so-called persister cells that have reduced their cellular activity and, hence, entered a state of multidrug tolerance (Lewis, 2010; Brauner et al., 2016). It is commonly assumed that a persister cell has undergone a phenotypic switch due to altered gene expression. However, whether a transient genetic change, as sometimes observed in the case of genetic heteroresistance (Andersson et al., 2019; Dewachter et al., 2019), could account for the persister phenomenon is currently unclear. At least two type I toxins, TisB and HokB, were suggested to affect the formation of persisters, either through PMF disruption, ATP depletion, or both (Dörr et al., 2010; Verstraeten et al., 2015; Berghoff et al., 2017a; Wilmaerts et al., 2018; Edelmann et al., 2021).

A recent debate in the field of persister research is whether and, if so, how TA systems affect the physiology of these special cells? This debate is particularly focused on type II TA systems and their role in persister physiology is highly questioned (Harms et al., 2017; Goormaghtigh et al., 2018), as also substantiated by retraction of some key publications (Maisonneuve et al., 2011, 2013). The debate is further fueled by the fact that genetic deletions of single TA systems do not generally result in persister-associated phenotypes, i.e., reduced survival rates upon antibiotic treatments when compared to wild-type strains. However, this does not necessarily reject TA systems as important factors for persistence. On the one hand, redundancy among TA systems might obscure phenotypes of single-deletion strains. On the other hand, phenotypes might only be detected under more complex experimental conditions, e.g., in infection models (Helaine et al., 2014; Ronneau and Helaine, 2019).

In the case of type I TA systems, the *tisB/istR-1* locus of *Escherichia coli* has been studied extensively with regard to persistence. Most type I TA systems are narrowly distributed among bacteria, and TisB homologs were only found in the family of *Enterobacteriaceae* (Coray et al., 2017). TisB was linked to persister formation more than 10 years ago (Dörr et al., 2010). Since transcription of toxin gene *tisB* is strongly activated upon DNA damage as part of the SOS response (Courcelle et al., 2001; Vogel et al., 2004; Berghoff et al., 2017b), the influence of *tisB* on persister formation was mainly elucidated using DNA-damaging antibiotics, such as the fluoroquinolone ciprofloxacin. Deletion of *tisB* significantly reduced persister levels upon treatment with ciprofloxacin, a finding that has since been interpreted as drug-induced persister formation caused by *tisB* expression (Dörr et al., 2010; Wagner and Unoson, 2012; Balaban et al., 2019). Even though a *tisB* related persister phenotype was validated independently using ciprofloxacin (Berghoff et al., 2017a), another study failed to observe a comparable phenotype when using the related antibiotic ofloxacin (Goormaghtigh and Van Melderen, 2019). Here, we would like to push forward the idea that toxin TisB, and probably other type I toxins, do not necessarily initiate the persister formation process, but mainly stabilize the persister state and affect the duration of cellular inactivity, both by primary and secondary effects. Our *Perspective* aims to stimulate a different view on the physiological role of type I toxins, and might help to solve some of the controversy concerning the link between TA systems and persistence in bacteria.

## Disruption of the PMF Is the Major Primary Effect of TisB in the Wild-Type Background

TisB has a length of 29 amino acids and integrates into the cytoplasmic membrane (Unoson and Wagner, 2008; Gurnev et al., 2012). Upon integration, TisB is believed to cause a breakdown of PMF, which is expected to deprive the cell of its foremost means to generate ATP (Unoson and Wagner, 2008; Gurnev et al., 2012; Edelmann et al., 2021). ATP depletion might ultimately link the action of TisB to growth inhibition and persister formation. While this hypothesis is apparently intuitive and can be corroborated for instance by chemical treatments with arsenate (Conlon et al., 2016; Shan et al., 2017), the evidence for TisB is mainly based on overexpression experiments (Unoson and Wagner, 2008; Edelmann et al., 2021), which might be a poor representative for *tisB* expression levels from its native locus. Furthermore, overexpression experiments often tend to result in drastic phenotypes that are, however, rarely further substantiated with experiments in the wild-type background. Therefore, we seek to review the material published on toxin TisB and present our view of what are likely authentic effects of toxin activity, and what effects are probably attributable to non-physiological expression levels in the experiment.

In point of fact, breakdown of the PMF (i.e., depolarization of the cytoplasmic membrane) by TisB is the effect best documented upon TisB expression from its native locus. In *E. coli* MG1655 wild-type cultures, TisB-dependent depolarization can be observed in a fraction of cells upon prolonged ciprofloxacin treatments (~20% depolarized cells after 4 h and ~50% after 6 h), and deletion of *tisB* almost completely prevents depolarization under these conditions (Berghoff et al., 2017a). Importantly, phenotypes of a *tisB* deletion, including lack of depolarization and a reduced persister level, can be observed only after several hours of antibiotic treatment in comparison to the wild type (Dörr et al., 2010; Berghoff et al., 2017a). These observations suggest that, in wild-type cultures, physiologically relevant amounts of TisB protein accumulate only after long periods of SOS induction, which is congruent with strong post-transcriptional repression of *tisB* mRNA by its 5′ UTR structure and antitoxin IstR-1 (Vogel et al., 2004; Darfeuille et al., 2007; Berghoff et al., 2017a).

TisB-dependent membrane depolarization is expected to ultimately affect ATP synthesis. Indeed, our unpublished results show that intracellular ATP levels are reduced in *E. coli* wild-type cultures after prolonged ciprofloxacin treatments, while ATP levels do not decrease in a *tisB* deletion strain. Interestingly, the decline of ATP only occurs at later time points and is rather moderate (~1.5-fold reduction after 4–6 h of ciprofloxacin). This suggests that drastic ATP depletion, as detected in response to strong TisB expression (Unoson and Wagner, 2008; Edelmann et al., 2021), does probably not resemble the wild-type situation. Alternatively, severe ATP depletion may only occur in a fraction of cells, which is not resolved in bulk measurements and demands single-cell reporters for ATP (Manuse et al., 2021). Either way, whether TisB-dependent depolarization and subsequent ATP depletion are the direct cause for drug-induced persister formation is yet to be demonstrated. Nevertheless, lowering the intracellular ATP levels can be an effective means to reach or modulate a persistent state (Conlon et al., 2016; Shan et al., 2017), and might be the mode of action for other type I toxins, such as HokB, which confers persistence by direct leakage of ATP (Wilmaerts et al., 2018).

## TisB Expression Causes Diverse Secondary Effects

Besides a drop in ATP levels as ultimate consequence of PMF disruption, further secondary effects can be assigned to TisB expression. For instance, TisB was shown to cause the formation of reactive oxygen species (ROS) upon high doses of ciprofloxacin and during long-term treatments (Edelmann and Berghoff, 2019). The mechanism that causes the formation of ROS is unknown. However, the ROS formed in response to TisB expression is mainly superoxide (Edelmann and Berghoff, 2019). Since superoxide is primarily formed as a byproduct of respiration (Imlay, 2013), it is tempting to speculate that the disruption at the cytoplasmic membrane caused by TisB generates an increased flux through the respiratory chain, which in turn leads to the formation of superoxide. Aerobic microorganisms are well adapted to deal with oxidative stress and have powerful enzymes to detoxify superoxide called superoxide dismutases (Imlay, 2013). Deletion of superoxide-scavenging enzymes revealed that they are essential for TisB-dependent persisters, while superoxide detoxification is of minor significance for persisters generated by other factors (Edelmann and Berghoff, 2019).

Early reports on the function of TisB indicated that overexpression of the toxin interferes with essential cellular processes such as replication, transcription, and translation (Unoson and Wagner, 2008). These findings were confirmed by more recent findings that link the expression of toxin TisB with the interference of essential cellular processes also in the wild-type background. In particular, protein biosynthesis was found to be impeded in a toxin-dependent manner in wild-type cells exposed to long treatments with ciprofloxacin (Edelmann et al., 2021). Indeed, comparison of wild-type cultures with a *tisB* deletion strain proves to be the most reliable when overexpression artifacts are to be avoided. Though phenotypes are typically rather subtle in toxin deletion mutants.

The link between TisB expression and protein biosynthesis is quite intriguing since a cessation of translation, either by toxin expression or bacteriostatic agents, might be a critical factor to confer persistence (Kwan et al., 2013; Cheverton et al., 2016; Rycroft et al., 2018). However, it is currently unknown whether TisB-dependent translation inhibition—and other secondary effects—influence the persister formation process.

## TisB Has the Potential to Stabilize the Persister State

**Figure 1:**
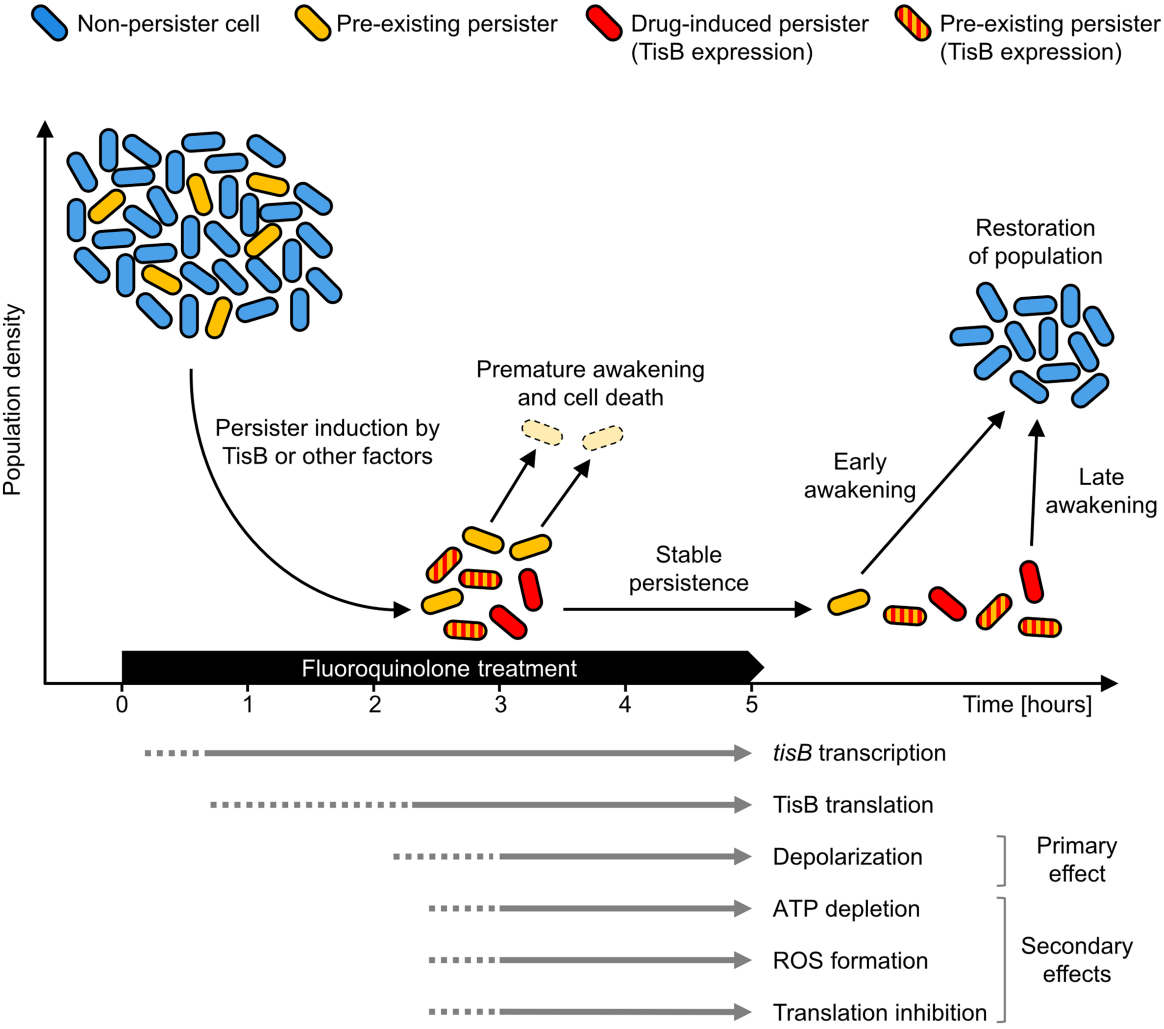
TisB-dependent stabilization of the persister state. Persister cells arise from exposure to a fluoroquinolone antibiotic (drug-induced persisters, red) from non-persister cells (blue) by expression of TisB or by other factors. Persisters may also exist before treatment (yellow). During treatment, accumulation of the TisB toxin has a stabilizing effect on the persister state. This probably affects both drug-induced persisters (red) and pre-existing persisters formed by other factors (striped red/yellow). Survival probability increases as the persister state is prolonged beyond the duration of antibiotic treatment. Non-stabilized persisters (yellow) have a higher probability for premature awakening and will be eliminated (pale yellow). Only cells that remain sufficiently long in the persister state can successfully contribute to population recovery after treatment. The arrows at the bottom represent key events as well as primary and secondary effects of TisB expression in *Escherichia coli* wild-type cultures. A solid line indicates mostly confirmed time points for an event, while a dotted line indicates that an event is likely to have already occurred at that time point but no corresponding data are available.

The *tisB* gene is one of the most highly induced genes of the SOS response, and transcripts can be detected within minutes after treatment initiation (Vogel et al., 2004; Berghoff et al., 2017b). As outlined above, post-transcriptional repression of *tisB* is expected to efficiently prevent immediate translation. Unfortunately, our attempts to directly detect TisB protein expressed from its native locus were not successful. We can, however, deduce some information from depolarization measurements. In wild-type cultures, depolarization is not observed after 2 h of ciprofloxacin treatment, whereas 20% of cells are depolarized after 4 h (Berghoff et al., 2017a), suggesting a strong delay in TisB translation and/or accumulation (Figure 1). Hence, only long-term treatments are relevant in order to observe TisB-dependent phenotypes. This certainly applies to depolarization as primary effect, but also to ATP depletion, ROS formation and translation inhibition as secondary effects (Edelmann and Berghoff, 2019; Edelmann et al., 2021).

The absence of measurable TisB-dependent effects at early time points does not necessarily challenge the assumption that the *tisB/istR-1* locus represents a regulatory module with a potential to induce the persister state. However, rapid TisB synthesis and subsequent TisB-dependent persister formation might be a rare event. It is rather plausible that—in most cells—the persister state is induced by other means, and that the actual function of TisB is more likely to be found at later time points. Therefore, we suggest a reinterpretation of TisB as a potential persistence-stabilizing factor. In such a scenario, TisB accumulation would take place in both pre-existing persisters and persisters that were formed in response to the fluoroquinolone treatment (drug-induced persisters) by the action of TisB or probably other factors (Figure 1). Upon prolonged treatment, TisB-dependent depolarization and subsequent secondary effects prevent premature awakening, thereby reducing the probability of a too early and thus lethal resumption of cellular activity (Figure 1). Furthermore, we assume that TisB accumulation is heterogeneous and differentially modulates wake-up kinetics. Indeed, wild-type cultures contain a subpopulation of late awakening persisters, which is not observed in a *tisB* deletion strain, when cultures are treated with a high dose of ciprofloxacin for 6 h (our unpublished results).

Our model predicts that, at early time points during fluoroquinolone treatments, wild-type and *tisB* deletion strains have comparable persister levels, but that persister levels deviate at later time points. Indeed, Dörr et al. (2010) observed in their original publication that lower persister levels of the *tisB* deletion strain can be obtained only if the ciprofloxacin treatment lasts longer than 3 h. This time point is in perfect accordance with our model in Figure 1.

## Concluding Remarks

Based on our observations with the *tisB* deletion strain, we suggest that future studies should aim to decipher the precise role of type I toxins in persister survival. However, we assume that, due to the inherent heterogeneity of populations, it might be difficult to differentiate clearly between an initiating and stabilizing function of toxins by experimental means. Recent efforts to study persister awakening on the single-cell level might offer a valuable starting point (Goormaghtigh and Van Melderen, 2019; Svenningsen et al., 2019; Windels et al., 2019; Kaplan et al., 2021).

It came to our attention that a persistence-stabilizing function of type I toxins might be in support of the “dormancy continuum hypothesis” (Ayrapetyan et al., 2015, 2018). Similarly to what was suggested for protein aggregation (Pu et al., 2019; Dewachter et al., 2021), accumulation of TisB and its concomitant effects might not only stabilize the persister state and affect dormancy depth, but also enable transition from a persister to a viable but non-culturable (VBNC) state. Further experiments are clearly needed to elucidate the role that type I toxins play in bacterial dormancy in response to environmental stress. Another intriguing question is whether a persistence-stabilizing function also applies to type II toxins. Using alternative experimental setups and focusing on long-term treatments might answer these questions in the future.

## Data Availability Statement

The data analyzed in this study is subject to the following licenses/restrictions: The datasets are unpublished results. Requests to access these datasets should be directed to bork.a.berghoff@mikro.bio.uni-giessen.de.

## Author Contributions

DE and BB wrote the manuscript. All authors contributed to the article and approved the submitted version.

## Funding

Work in the group of BB was supported by the German Research Council (DFG) in the framework of the SPP 2002 (BE 5210/3-1 and BE 5210/3-2) and by the University of Giessen (Research Grant).

## Conflict of Interest

The authors declare that the research was conducted in the absence of any commercial or financial relationships that could be construed as a potential conflict of interest.

## Publisher’s Note

All claims expressed in this article are solely those of the authors and do not necessarily represent those of their affiliated organizations, or those of the publisher, the editors and the reviewers. Any product that may be evaluated in this article, or claim that may be made by its manufacturer, is not guaranteed or endorsed by the publisher.
